# Suhuang Zhike Capsules for the Treatment of Cough Variant Asthma: A Meta-analysis

**DOI:** 10.1155/2020/9485746

**Published:** 2020-12-31

**Authors:** Cheng Gu, Wenpan Peng, Zhichao Wang, Yong Xu, Di Han, Xianmei Zhou

**Affiliations:** ^1^Affiliated Hospital of Nanjing University of Chinese Medicine, Nanjing 210029, China; ^2^Department of Respiratory Medicine, Jiangsu Province Hospital of Chinese Medicine, Affiliated Hospital of Nanjing University of Chinese Medicine, Nanjing, Jiangsu 210029, China

## Abstract

Cough variant asthma (CVA) is a unique type of asthma characterized by cough as the only or primary clinical presentation. Inhaled glucocorticoid is the main treatment in clinical practice currently, but its efficacy remains relatively unsatisfactory. Traditional Chinese medicine has certain advantages in the treatment of CVA, and at present, the most commonly used traditional Chinese medicine is Suhuang Zhike Capsule (SZC). The aim of this study was to systematically evaluate the efficacy and safety of SZC in the treatment of CVA using a meta-analysis. A comprehensive search of papers published in the PubMed, Embase, Cochrane Library, China National Knowledge Infrastructure (CNKI), Chinese Biomedical Literature database (CBM), Wanfang Database, and VIP Information (VIP) from January 2018 to June 2019 was conducted. Review Manager 5.3 was used to carry out a meta-analysis of 10 studies that fulfilled the inclusion criteria. In a total of 10 randomized controlled trials, 896 CVA patients were included. The results showed the following: (1) compared with conventional Western medicine, SZC can effectively increase the efficacy rate of CVA (RR 1.25, 95% CI, 1.16–1.35, *P* < 0.00001) and (2) compared with other traditional Chinese medicines, SZC can effectively increase the efficacy rate of CVA (RR 1.44, 95% CI, 1.01–2.05, *P*=0.05), In conclusion, our study builds on existing clinical evidence showing that SZC is safe and effective in treating CVA. However, larger randomized controlled trials are required for further validation.

## 1. Introduction

Cough variant asthma (CVA) is a unique type of asthma that is characterized by cough as the only or primary clinical presentation. Most CVA patients do not show obvious signs of wheezing and shortness of breath and only have airway hyperresponsiveness. CVA mostly occurs at night or dawn and presents as a chronic, recurrent, and irritating dry cough that is exacerbated when hot/cold stimuli or smoke/foul smells are present [1]. Although CVA is not life threatening, its tendency to occur or exacerbate at night causes a lot of disruption to patients' lives. In addition, CVA tends to progress to classical asthma [2, 3]. Both CVA and classical asthma share a similar pathogenesis involving the combined interaction between genetics, immunity, and environment and is mostly associated with airway inflammation [4], airway hyperresponsiveness [5–7], and airway remodeling [8, 9].

The principles for treating CVA are identical to classical asthma [10], and the recommended treatment guidelines are inhaled glucocorticoids combined with bronchodilators for, at least, 8 weeks [10], with some patients requiring longer term of treatment. Low oral doses of glucocorticoids are recommended for patients with severe airway inflammation or poor response to inhaled glucocorticoids. Leukotriene receptor antagonists can be used for a minority of patients that do not respond to inhaled glucocorticoids [11]. However, in consideration of the side effects of long-term usage of glucocorticoids, new treatment options are necessary.

Professor Enxiang Chao, one of the most famous specialists of traditional Chinese medicine, believed that the etiological mechanism of CVA is lung invasion by wind evil, lung qi obstruction, and airway contracture [12]. He developed SZC in order to clear the lungs and relieve coughing and to treat CVA in clinical practice in China. SZC is composed of *Ephedra sinica Stapf*, Beefsteak plant leaves and seeds, earthworms, loquat leaves, *Periostracum cicadae*, *Peucedanum praeruptorum Dunn* roots, Greater burdock seeds, and five-flavor berries. Modern pharmacological experiments showed that Beefsteak plant leaves, *Ephedra sinica Stapf*, and *Periostracum cicadae* in SZC have anti-inflammatory, antitussive, and antiwheezing effects [13–16]. Currently, there are an increasing number of clinical trials using SZC to treat CVA, but most are small randomized controlled trials (RCTS) with small sample sizes, making it difficult to draw reliable conclusions. The aim of this meta-analysis is to assess the efficacy of SZC in the treatment of CVA from data collected in RCTs.

## 2. Materials and Methods

### 2.1. Publication Search Strategy

A search of papers published in the PubMed, Embase, Cochrane Library, China National Knowledge Infrastructure (CNKI), Chinese Biomedical Literature database (CBM), Wanfang Database, and VIP Information (VIP) from January 2018 to June 2019 was conducted. Two reviewers (Cheng Gu and Wenpan Peng) used the following keywords or free text terms to independently search papers in these electronic databases. The term used were “Suhuang Zhike capsules,” “allergic cough,” “variant cough,” and “cough asthma” in Chinese and “cough variant asthma” and “cough-variance asthma” in Chinese and English. The authors of significant publications or experts in the relevant field were contacted for potential studies. Also, a search of unfinished research in the Cochrane central register of controlled trials, National Research Register (NRR), and clinical controlled trials (CTT) was conducted. A search of conference papers in ISTP, ISI proceedings, and OCLC Firstsearch proceedings was conducted. A search of the grey literature in GreyNet, Database of Abstracts of Reviews of Effects (DARE), and System for Information on Grey Literature in Europe (SIGLE) was conducted.

### 2.2. Inclusion Criteria

Studies to be included in a meta-analysis needed to meet all of the following criteria: (a) type of study: the type of the study needed to be RCTs which may have “RCT” in the title, abstract, or methods, its methods should have used a control group, randomization, allocation concealment, and all of the studies needed to be published from 1 January 2008 to 30 June 2019, (b) study subjects: patients with a definitive diagnosis of CVA of both genders and age >18 years, and (c) Intervention experiment: SZC only was used for the treatment group, and other drugs, placebo, or blank control were used for the control group.

### 2.3. Exclusion Criteria

The following studies were excluded: (a) SZC combined with other drugs were used for the treatment group in the original study, (b) studies in which the age of the study subjects was <18 years, and (c) republished papers, of which only one paper was used.

### 2.4. Markers

The primary prognostic markers were as follows: (a) Overall efficacy rate: clinical remission rate + marked improvement rate + improvement rate, (b) traditional Chinese medicine syndrome overall efficacy: clinical remission rate + marked improvement rate + improvement rate, (c) bronchial provocation test negativity conversion rate, and (d) adverse reactions.

The clinical efficacy for traditional Chinese medicine was classified as clinical remission, marked improvement, improvement, and not effective according to guidelines for clinical studies on new traditional Chinese medicine (Table 1) [17]. The nimodipine method was used to assess the efficacy for traditional Chinese medicine symptoms [18]. Efficacy was classified as clinical remission, marked improvement, improvement, and not effective (Table 2).

### 2.5. Study Selection and Data Extraction

The following information was collected from each study: (1) basic information, such as the first author's name and year of publication, (2) number of participants in each group and their gender, age, and medication duration, and (3) intervention measures and results of each experiment. The methodological quality of these studies was assessed by the two reviewers according to Cochrane Handbook for Systematic Reviews of Interventions 5.1.0, including (1) sequence generation, (2) allocation hiding, (3) blind method, (4) incomplete outcome data, (5) alternative outcome report, and (6) other sources of bias. Two researchers were responsible for independent screening and data extraction. Firstly, they read the title and abstract before reading the entire text of relevant papers. Next, cross validation was carried out by way of a third researcher who was responsible for judging and discussing the study when there were differences between the two researchers. The content of the data extracted included authors, year of publication, sample size, age and gender of subjects, detailed methodological information, detailed information on intervention measures, results, and adverse reactions.

### 2.6. Quality Evaluation

Two researchers assessed the methodological quality of the RCTs. The risk of the bias assessment tool recommended by the Cochrane manual was used to assess the risk of bias in the included papers. The content mainly included the randomization method, whether allocation was concealed, whether subjects and investigators were blinded, whether result assessors were blinded, whether results data were intact, and whether selective reporting was present. The actual situation of the included papers was then used to classify papers as low risk, high risk, and unclear.

### 2.7. Data Analysis

Review Manager 5.3 (Cochrane Collaboration, Oxford, United Kingdom) was used to combine data for meta-analysis. The combined RR with 95% CI was calculated to compare binary and continuous variables. If heterogeneity was present in the combined studies, the random effects model (I 2 > 50％) was used; otherwise, the fixed effects model was used. A difference of *P* < 0.05 was considered to be statistically significant. If there were more than 10 studies included in the meta-analysis, Stata 14.0 was used for the Egger test and Begg test to detect publication bias.

## 3. Results

### 3.1. Search Results

**Figure 1:**
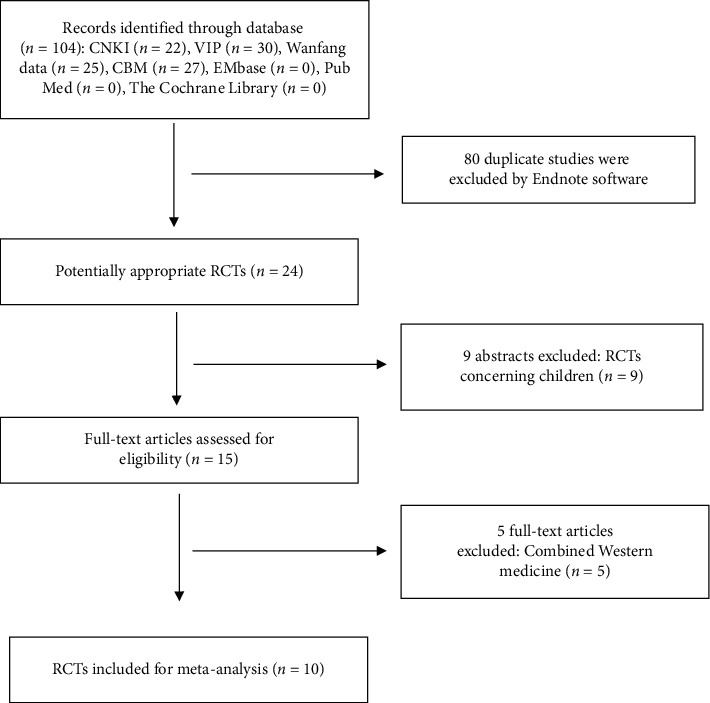
Flow diagram of the literature search process.

One hundred and four potentially related papers were selected based on the predefined search criteria, of which 22, 30, 25, and 27 papers were from the CNKI, VIP, Wanfang database, and CBM, respectively. Following that, EndNote X8 was used to exclude 80 repeated papers. After reading the title and abstract, 9 papers involving children were excluded. Following that, 2 reviewers read the full text of the remaining 15 papers, and 5 papers involved combinational drugs and were, therefore, excluded. Therefore, a total of 10 qualified trials were used for the current meta-analysis. A total of 896 patients participated in these 10 studies, of which 535 were from the SZC group and 361 were from the control group (Figure 1 and Table 3).

### 3.2. Methodological Evaluation of the Included Studies

**Figure 2:**
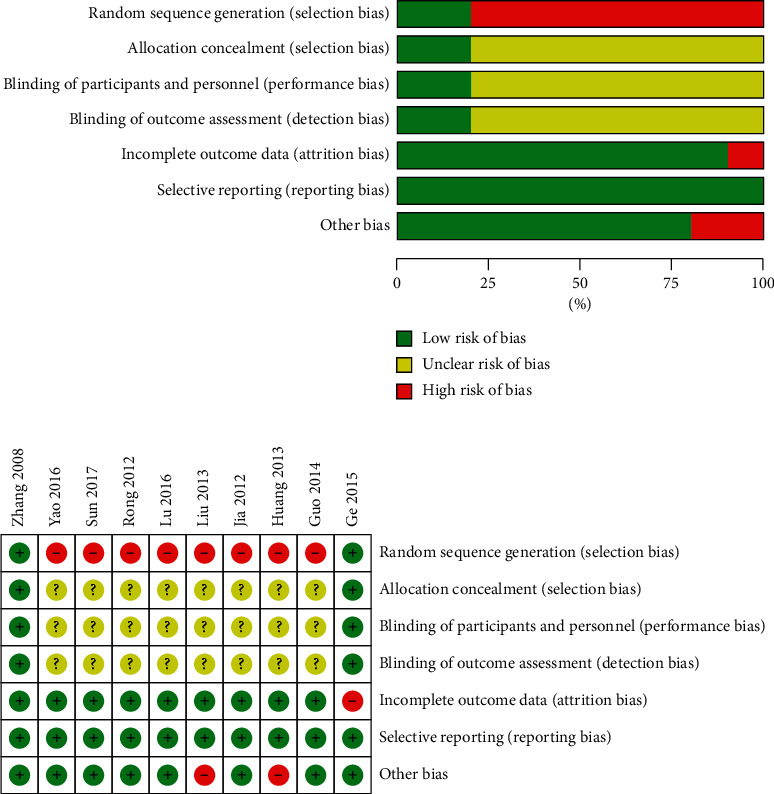
Risk of bias graph and risk of bias summary.

All included studies mentioned randomization, but only 2 studies [19, 20] described the specific randomization method, and 2 papers [19, 20] mentioned allocation concealment and blinding. The remaining studies [21–28] did not mention randomization and blinding, and we were unable to determine whether selective reporting bias was present. A total of 3 trials [19, 20, 23] mentioned the shedding and removal of cases, none of which were lost to follow-up, while the rest [21, 22, 24–28] were not mentioned, but their results were complete and other biases were unclear.  Figure 2 lists the detailed information on the methodological quality of all included studies.

### 3.3. Meta-Analysis of SZC in the Treatment of Cough Variant Asthma

#### 3.3.1. Efficacy Rate

**Figure 3:**
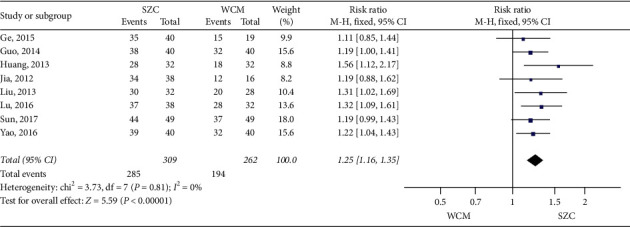
The efficacy rate using SZC versus conventional Western medicine.

**Figure 4:**
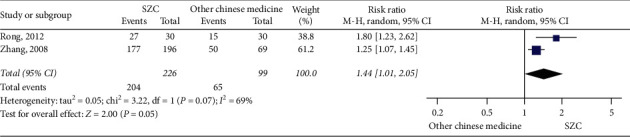
The efficacy rate using SZC versus other traditional Chinese medicine.

(1) Comparison of SZC with conventional Western medicine: as shown in Figure 3, 8 studies examined the differences in the efficacy rate between SZC and Western medicine [19, 21–25, 27, 28]. There were 571 patients included in total, including 262 in the control group and 309 in the treatment group. As there was no heterogeneity in the 8 studies (chi-square = 3.73, *P*=0.81, I^2^ = 0%), the fixed effects model was used for statistical analysis. The results of the study showed that compared with conventional Western medicine, SZC can effectively increase the efficacy rate (RR 1.25, 95% CI: 1.16–1.35, *P* < 0.00001) (Figure 3).

(2) Comparison of SZC with other traditional Chinese medicines: as shown in Figure 4, 2 studies examined the differences in the efficacy rate between SZC and Western medicine [20, 26]. There were 325 patients included in total, including 99 in the control group and 226 in the treatment group. As there was heterogeneity in the 2 studies (chi-square = 3.22, *P*=0.07, I^2^ = 69%), the random effects model was used for statistical analysis. The results of the study showed that compared with other traditional Chinese medicine, SZC can effectively increase efficacy (RR 1.44, 95% CI: 1.01–2.05, *P*=0.05) (Figure 4).

#### 3.3.2. Traditional Chinese Medicine Symptom Efficacy Rate

**Figure 5:**
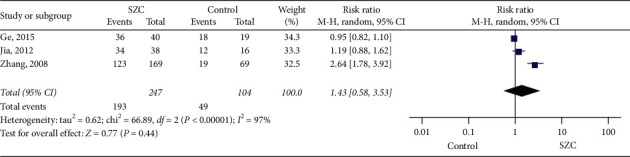
Traditional Chinese medicine symptom efficacy rate.

Three studies examined the traditional Chinese medicine symptom efficacy rate [19, 20, 23]. There were 351 patients included in total, including 104 in the control group and 247 in the treatment group. Since heterogeneity was shown in three studies (chi-square = 66.89, *P* < 0.00001, I^2^ = 97%), the random effects model was used for statistical analysis. Results showed that there was no significant difference between the SZC and control groups (RR 1.43, 95%  CI:0.58–3.53. *P* < 0.00001) (Figure 5).

#### 3.3.3. Bronchial Provocation Test Negativity Conversion Rate

**Figure 6:**
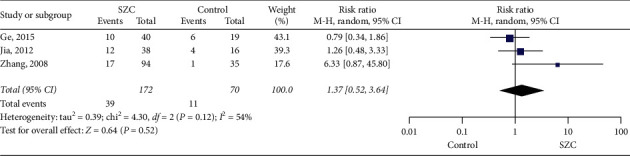
Bronchial provocation test negativity conversion rate.

Three studies examined the bronchial provocation test negativity conversion rate [19, 20, 23]. There were 242 patients included in total, including 70 in the control group and 172 in the treatment group. Since heterogeneity was shown in 3 studies (chi-square = 4.30, *P*=0.12, I^2^ = 54%), the random effects model was used for statistical analysis. Results showed that there was no significant difference between the SZC and control groups (RR 1.37, 95%  CI:0.52–3.64. *P*=0.52) (Figure 6).

#### 3.3.4. Adverse Reactions

Only 1 patient developed nausea and vomiting after taking SZC, and no other adverse reactions were reported [28]. The common adverse reactions of Western medicine or traditional Chinese medicine in the control group were headache, dizziness, drowsiness, fatigue, dry mouth, muscle tremor, accelerated heart rate, and oral ulcer. However, some of the literature do not specify the number of adverse events reported, it is difficult to calculate the total incidence of adverse reactions.

#### 3.3.5. Publication Bias Analysis

**Figure 7:**
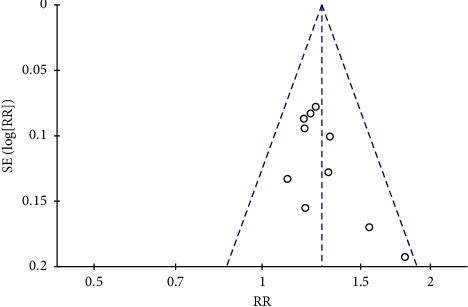
Funnel plots for assessing publication bias.

As shown in Figure 7, funnel plot analysis was carried out using the efficacy rate. The funnel plot shows that poor symmetry and publication bias may be present. Stata 14.0 was used for Egger and Begg tests on the 10 studies, which showed that publication bias was absent (*P*=0.072, *P*=0.210, respectively) (Figure 7).

## 4. Discussion

The present study describes a meta-analysis of RCTs investigating the efficacy of SZC to treat CVA. A total of 10 randomized controlled trials were included in this study, including 896 CVA patients. Results showed that the efficacy rate of SZC in the treatment of CVA is superior to conventional Western medicines and other traditional Chinese medicines, and the incidence of adverse reactions is lower than that in the control groups.

Our study confirms the results of Zhang et al. [29], which showed that SZC combined with Western medicine had a significant effect on CVA, suggesting that SZC might have a positive effect on CVA treatment. In terms of adverse reactions, SZC had fewer adverse reactions than salmeteroticasone combined with montelukast [30], suggesting its relative safety. However, there are some limitations of this study, suggesting that further research is required. First, the sample sizes of the RCTs included were very small, and it is difficult to rule out the influence of contingency factors. The clinical trial protocols of most of the included studies were still not stringent enough; most used symptom markers, and few studies used eosinophil counts, which means it is not objective enough. The reasons for heterogeneity may include the following three points: first, the drugs in the control group were inconsistent, some were inhaled and some were oral Chinese medicine; second, the treatment course was different, some were 14 days and some were 28 days; and third, the methodology might be different, such as some used blinding and some did not use blinding. In addition, the overall methodological quality of the RCTS included was not high; for example, only 3 studies described randomization and allocation concealment, while only 3 studies described blinding. In addition, follow-up was not described in the included studies, and there was no long-term efficacy evaluation of SZC in the treatment of CVA.

## 5. Conclusions

The current evidence shows that SZC is effective in treating CVA. However, due to the limitations of this study, we recommend that future traditional Chinese medicine clinical trials should be conducted as standardized, multicenter, and large sample size high-quality randomized, double-blind trials to more accurately assess its efficacy and safety.

## Figures and Tables

**Table 1:** Traditional Chinese medicine clinical efficacy and its description.

Group	Description
Clinical remission	Complete remission of cough symptoms and scores of all primary symptoms were zero
Marked improvement	Coughing significantly alleviated or disappears. The scores of all primary symptoms decreased by 2 grades
Improvement	Coughing significantly alleviated. The scores of all primary symptoms decreased by 1 grade, or the score of 1 symptom decreased by 2 grades while another symptom decreased by 1 grade
Not effective	Coughing was not significantly alleviated or worsened.

**Table 2:** Determination criteria for traditional Chinese medicine symptoms overall efficacy and its description.

Group	Description
Clinical remission	*n* ≥ 95%
Marked improvement	70% ≤ *n* <95%
Improvement	30% ≤ *n* <70%
Not effective	*n* < 30%

Efficacy index (*n*) = (pretreatment score–posttreatment score)/pretreatment score × 100%. 0 points: no cough, 3 points: occasional coughing, 6 points: frequent coughing, 9 points: persistent coughing.

**Table 3:** Characteristics of studies conforming to criteria.

Included studies	N		Intervention measures		Treatment course	Results
	T	C	T	C		
Zhang, 2008	196	69	SZC	Zhike Ningsou capsules	14d	①②④⑥⑦
Rong, 2012	30	30	SZC	Compound liquorice tablets	14d	①
Jia, 2012	38	16	SZC	Fluticasone/salmeterol inhalation powder	28d	①②③④⑤⑥⑦
Huang, 2013	32	32	SZC	Montelukast sodium	14d	①
Liu, 2013	32	28	SZC	Ketotifen	7–14d	①⑦
Guo, 2014	40	40	SZC	Salbutamol nebulization solution	14d	①⑦
Ge, 2015	40	20	SZC	Fluticasone/salmeterol inhalation powder	28d	①②③④⑤⑥⑦
Yao, 2016	40	40	SZC	Montelukast sodium	14d	①
Lu, 2016	38	38	SZC	Montelukast sodium	14d	①②④
Sun, 2017	49	49	SZC	Montelukast sodium	14d	①⑦

Results: ①overall efficacy for cough; ②traditional Chinese medicine overall efficacy; ③traditional Chinese medicine syndrome overall efficacy; ④eosinophil count; ⑤serum IgE level; ⑥bronchial provocation test negativity conversion rate; ⑦safety analysis.

## Data Availability

The extracted data used to support the findings of this study are available from the corresponding author upon request.
